# Brown tumor of the knee as the first presentation of primary hyperparathyroidism caused by parathyroid adenoma: A case report

**DOI:** 10.1016/j.radcr.2023.01.080

**Published:** 2023-03-06

**Authors:** Vaishnavi C Tapadia, Romana Riyaz, Abhigan Babu Shrestha, Javeed Akhtar Ankolvi

**Affiliations:** a Department of Radiology, Shadan Institute of Medical Sciences and Research, Hyderabad, Telanhana, India; b M Abdur Rahim Medical College, Dinajpur, Bangladesh

**Keywords:** Brown tumor, Hyperparathyroidism, Parathyroid hormone, Adenoma

## Abstract

Brown tumor of the knee is a focal benign cystic lesion of bone. The etiopathogenesis of brown tumor is believed to be abnormal bone metabolism in patients with hyperparathyroidism. We present a case of a 32-year-old male with recurrent knee pain, weakness of the lower limb and a nodular mass on left inferior lobe of thyroid. Timely identification of the underlying cause and localization of lesion(s) is important because the management and prognosis vary based on the etiology. The diagnosis of a brown tumor is the result of the sum of the medical history of patients with clinical, radiographic imaging, histopathological, hematological, and biochemical laboratory investigations.

## Introduction

A brown tumor also known as osteitis fibrosa cystica is a unique manifestation of reactive focal bony lesion resulting from rapid osteoclastic activity either due to excessive secretion of parathormone (PTH) or paraneoplastic syndrome. The incidence is less than 5% in primary hyperparathyroidism and is more commonly seen in secondary hyperparathyroidism [1]. Common sites of brown tumors are the facial bones, jaws, ribs, clavicle and pelvic girdle and the lower limb is rarely affected [2]. Brown tumor is a misnomer; and is not the result of a neoplastic process as the name suggests. Its name derives from the fact that there is a characteristic reddish-brown coloration due to hemorrhagic foci, hemosiderin deposition and highly fibro-vascularized tissue replacing the normal bony tissue into the fluid-filled cysts as a result of osteolysis. The clinical symptoms of brown tumors vary with the size and site of the lesion and are nonspecific. It may present with complaints of swelling, pathologic fracture, and bone pain [3]. In this article, we present an unusual case of brown tumor of the knee in a 32-year-old male with primary hyperparathyroidism caused by parathyroid adenoma and a literature review of brown tumor of the knee, its etiopathogenesis, clinical features, radiological criteria and mode of management.

## Case presentation

We present a case of a 32-year-old Indian male admitted to the orthopedics department. The patient had a history of recurrent pain in the right knee for the past 4 months. Mechanical diffuse pain was exacerbated during weight bearing and walking and decreased while resting and subsided on taking analgesics. He was on a Vitamin D3 30,000 IU supplement course for 6 weeks. Pain and lower limb motor weakness were the only musculoskeletal complaints. His other systemic medical history was unremarkable. The range of motion of the knee was normal. He had no history of trauma. On physical examination of the knee, tenderness or edema, or external abnormality was not observed. While a nodule was felt in the inferior lobe of the left thyroid.

Laboratory analysis showed an elevated serum calcium level of 7.8 mmol/L (reference range, 2.1-2.6 mmol/L) and elevated serum alkaline phosphatase level of 590 IU/L (reference range, 50-240 IU/L) and a low serum phosphate level of 0.54 mg/L (reference range: 0.77-1.32 mg/L) and low total vitamin D10.6 ng/ml(reference range:21-100). Subsequent testing revealed a high PTH level of 138 pmol/L (reference range: 1.2-5.8 pmol/L). Rheumatoid factor, C- reactive protein and Anti cyclic citrullinated peptide antibody, thyroid and other renal function tests were within the normal range.

Due to inconclusive test results and continued pain in the right knee and nodule as palpatory finding, the patient was advised for further imaging investigations.

**Fig. 1(A):**
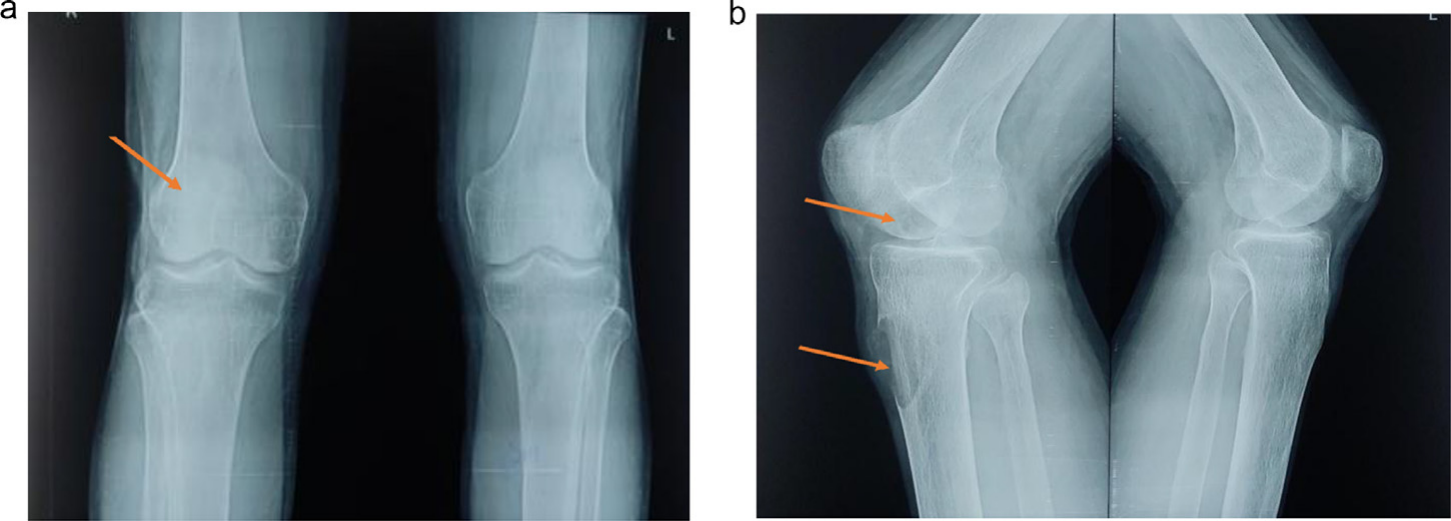
Simple radiograph of bilateral knee joints presenting a lesion in the right knee on Anteroposterior view while the left knee is normal. (B) A well-defined osteolytic geographic lesion at the epiphysis of the medial femoral condyle extending up to the subarticular region and an eccentric lytic lesion at the proximal end of the tibia showing cystic changes with decreased bone density.

On simple radiographs of the bilateral knee joints, the cortical bone of the right distal end of femur showed well defined osteolytic geographic lesions at the epiphysis of medial femoral condyle extending upto the subarticular region. An eccentric lytic lesion with rim-like margins at the proximal end of the tibia showed cystic changes with decreased bone density and the trabecular bone of the upper end of tibia was thinned while the left knee was normal. (Figs. 1A and B)

**Fig. 2:**
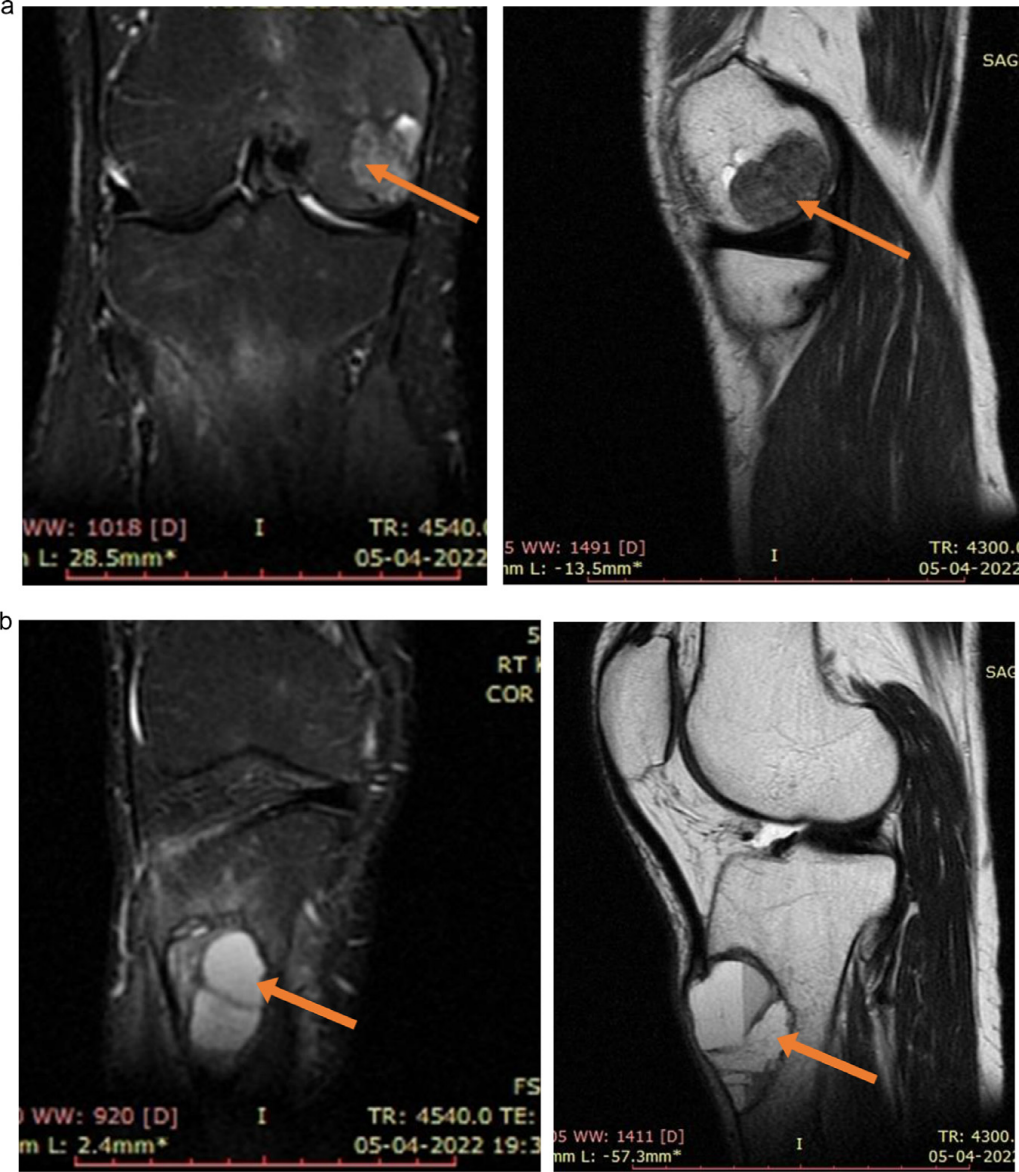
(A) MRI of the right knee shows a well-defined heterogeneously hyper-intense lesion on Short-TI Inversion Recovery and hypo intense on T2 with small T2 hyperintense foci in the epiphyseal region of medial femoral condyle with subarticular extension. (B) Another eccentric multiloculated fluid signal intensity lesion with fluid - fluid levels which are hyper intense on T2 and Short-TI Inversion Recovery noted in the upper end of the tibia with moderate marrow edema.

Magnetic resonance imaging (MRI) was performed on the part showing cystic bone change to determine the bone tumor. A well-defined heterogeneously hyper-intense lesion on Short-TI Inversion Recovery and hypointense on T2W1 with T2W1 hyperintense foci in the epiphyseal region of medial femoral condyle with subarticular extension. Another eccentric multiloculated fluid signal intensity lesion with fluid-fluid levels which are hyper-intense on T2W1 and Short-TI Inversion Recovery noted in the upper end of the tibia with moderate marrow edema was observed (Fig. 2). Based on the MRI findings, a brown tumor was suspected.

**Fig. 3:**
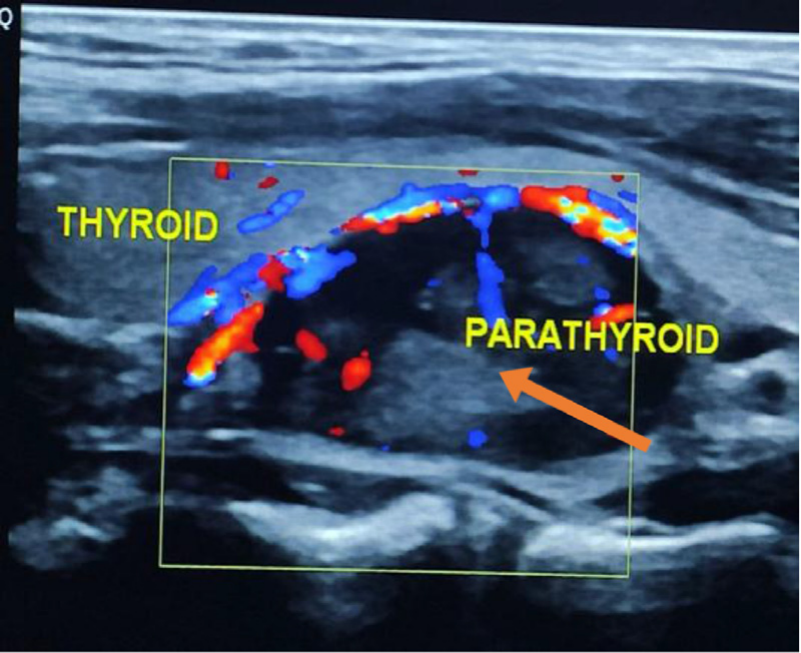
Neck ultrasound images show a hypoechoic, well-circumscribed mass with color-Doppler of monopolar vascular pattern referred to “as the arc or rim sign,” located posteriorly inferiorly to the lower pole of the left thyroid lobe.

**Fig. 4:**
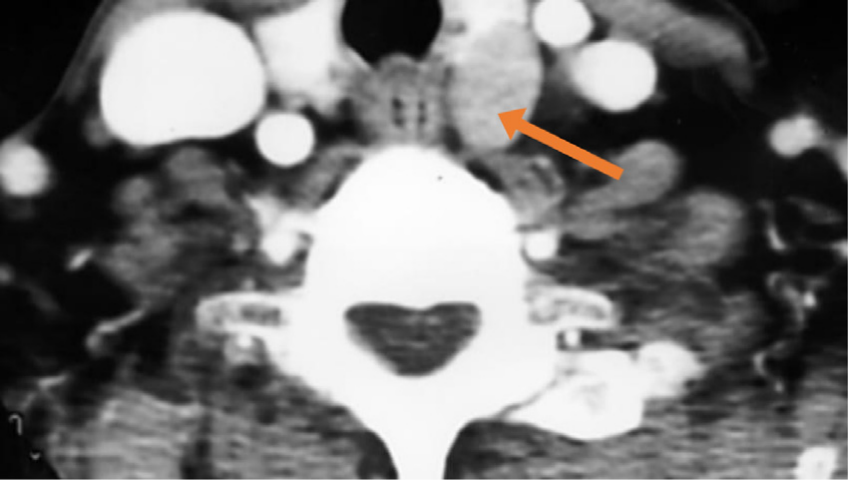
Contrast CT scan of the neck showing a parathyroid adenoma posterior to the left lobe of the thyroid.

Furthermore, neck ultrasound images shows a solid, hypoechoic, well circumscribed mass with color-Doppler of monopolar vascular pattern was located posteroinferiorly to the lower pole of left thyroid lobe, these ultrasound features were typically suggestive of the parathyroid adenoma (Fig. 3) and contrast CT scan of the neck shows a parathyroid adenoma posterior to the left lobe of the thyroid. (Fig. 4)

To arrive at a conclusive diagnosis, a histopathological study of the tibial lesion was performed and revealed the presence of a giant cell granuloma with microscopic features of increased osteoclasts activity, hemosiderin pigment deposition and bone resorption suggestive of hyperparathyroidism.

Hence, the high level of PTH and parathyroid mass on imaging findings has raised the possibility of a brown tumor of the knee. Later, an opinion was sought from the general surgeon who then advised and performed a left-sided parathyroidectomy. Since the patient was lost to follow-up, subsequent changes in the lesion of the tumor are not known.

## Discussion

Clinically brown tumor of the knee in the setting of primary hyperparathyroidism is very rare. There has been only 1 report on a brown tumor of the knee by primary hyperthyroidism in the medical literature [4]. Primary hyperparathyroidism is an endocrine disease that results from excessive production of the parathyroid hormone (PTH) and causes destructive changes in the balance of osteoblasts and osteoclasts through activation of the osteoclast-enhancing bone catabolism. Here, bleeding and repetitive granulation of tissue partially develops in the bone and gradually fill with fibrous tissues which rapidly proliferate. These changes of bone either as uni- or multifocal bone lesions are observed in approximately 10%-20% of patients with long-standing-hyperparathyroidism and are referred to as a brown tumors [5]. The main effects of PTH are to increase the level of plasma calcium by increasing the release of calcium and phosphate from the bone matrix, increasing calcium reabsorption by the kidneys, and increasing renal production of 1,25-dihydroxy vitamin D3 (calcitriol), which in turn increases intestinal absorption of calcium. Increased levels of serum calcium and parathyroid hormones and reduced levels of serum phosphate as well as decreased total vitamin D observed in this patient were sufficient to diagnose primary hyperparathyroidism. Ultrasound and CT scan techniques can be used also to detect the diseased parathyroid gland. The main causes of this condition are solitary adenoma in 80%-85% of the patients, multiple adenomas in 5% of the patients, and parathyroid hyperplasia in 10%-15% [6]. In our case, the tumor involves the right knee articulation; medial femoral condyle, patella and tibial tuberosity. Radiological features (cyst-like radiolucency) shown by other skeletal lesions, the diagnosis can be difficult. Histology cannot assure a certain diagnosis, some lesions, such as giant cell tumors or granuloma, aneurysmal bone cysts and cherubism, show similar macroscopic and microscopic features [7] more challenging to confirm the diagnosis of brown tumor [7]. Differential diagnosis is possible only by complete evaluation of clinical, radiological and biochemical evidence. Clinical history, especially similar history of endocrine familial disorders is crucial, our patient denied a family history of hyperparathyroidism or other endocrine disorder. A screen for multiple endocrine neoplasia types 1 and 2A must be performed, including serum prolactin, insulin-like growth factor-1, 24-hour urine vanillylmandelic acid and free cortisol levels. Brown tumors appear in around 10% of the cases in the advanced stages of chronic kidney disease [8]. Our patient's renal function tests were in the normal range. Imaging findings of the entire skeletal system is essential to diagnose other bony lesions. MRI is a useful imaging modality to detect solid and cystic components of the tissue [2]. In our patient bony cystic lesion was observed only in the knee and no other bony site had a similar lesion. The management of a brown tumor depends on the severity of the lesion present and treatment of hyperparathyroidism is the first step. Parathyroidectomy is the treatment of choice, several studies have demonstrated the normalization of the biochemical aspects of the disease and the regression of skeletal abnormalities promptly [3,9]. Severe untreated primary hyperparathyroidism results in fracture, renal failure, secondary hemorrhage, and multiple brown tumors [10]. Therefore, it is highly essential to diagnose or differentiate brown tumors from other musculoskeletal tumors at an early stage, and as soon as possible.

## Conclusion

In conclusion, physicians should keep in mind brown tumors during daily practice as a differential diagnosis. Timely identification of the underlying cause and localization of lesion(s) is important because the management and prognosis vary based on the etiology and the site of the lesion and in the case of hypercalcemia and radiographic evidence of lytic lesion, primary hyperparathyroidism should always be kept in differential diagnosis and should be looked into once more common causes.

## Ethical approval

None.

## Provenance and peer review

Not commissioned, externally. Peer reviewed.

## Patient consent

Written informed consent was obtained from the patient for the publication of this case report and accompanying images. A copy of the written consent is available for review by the Editor-in-Chief of this journal on request.
